# Draft Genome Sequence of *Clostridium* sp. Strain W14A Isolated from a Cellulose-Degrading Biofilm in a Landfill Leachate Microcosm

**DOI:** 10.1128/genomeA.00985-16

**Published:** 2016-09-22

**Authors:** Emma Ransom-Jones, James E. McDonald

**Affiliations:** School of Biological Sciences, Bangor University, Bangor, United Kingdom

## Abstract

Here, we report the draft genome of *Clostridium* sp. strain W14A, isolated from the anaerobic, cellulolytic biofilm of a cotton string sample incubated in a landfill leachate microcosm. The draft genome comprises 131 contigs, 3,823,510 bp, 51.5% G+C content, and 4,119 predicted coding domain sequences.

## GENOME ANNOUNCEMENT

Members of the genus *Clostridium* have been consistently detected as abundant members of the landfill microbial community (1–4), but few species have been isolated from this environment. The majority of landfill clostridia contribute to the hydrolysis of cellulose that represents the most abundant biodegradable component of landfill waste (1, 2).

*Clostridium* strain W14A was isolated from dewaxed cotton string incubated in a landfill leachate microcosm previously described by McDonald et al. (3), via the anaerobic roll tube method of Hungate (5), using modified M2GSC broth medium as described by Miyazaki et al. (6) with rumen fluid omitted. DNA extraction was performed using the Wizard Genomic DNA purification kit (Promega) and quantified using a Qubit Florometer (Life Technologies). Genome sequencing was performed by MicrobesNG (Birmingham) with the genomic DNA library prepared using the Nextera XT library prep kit (Illumina) following the manufacturer’s protocol with the following modifications: 2 ng of DNA instead of 1 were used as input, and PCR elongation time was increased to 1 min from 30 s. DNA quantification and library preparation were carried out on a Microlab STAR automated liquid handling system (Hamilton). Libraries were sequenced on the Illumina HiSeq using a 250 bp paired end protocol.

Reads were adapter trimmed using Trimmomatic 0.30 with a sliding window quality cutoff of Q15 (7) and *de novo* genome assembly was carried out with SPAdes (version 3.7) (8) via MicrobesNG (Birmingham). Annotation was performed via the RAST server (version 2.0) (9). The draft genome comprises 131 contigs, 3,823,510 bp and 51.5% G+C content as determined by RAST (9). Genome comparison via RAST (9) and 16S rRNA gene analysis (data not shown) suggests that *Clostridium* sp. strain W14A is a novel species most closely related to *Clostridium leptum* DSM 753. The genome contains 4,119 predicted coding domain sequences and 366 subsystems, and includes genes involved in sucrose, maltose, lactose and galactose utilization, and spore formation.

### Accession number(s).

This whole-genome shotgun project has been deposited at DDBJ/ENA/GenBank under the accession no. MBSV00000000. The version described in this paper is the first version.
